# Gallic acid reverses ovarian disturbances in mice with letrozole-induced PCOS via modulating Adipo R1 expression

**DOI:** 10.1016/j.toxrep.2022.10.009

**Published:** 2022-10-20

**Authors:** Mohd Zahoor ul haq Shah, Meenakshi Soni, Vinoy kumar Shrivastava, Manzoor Ahmad Mir, Showkeen Muzamil

**Affiliations:** a Laboratory of Endocrinology, Department of Bioscience, Barkatullah University Bhopal, India; b Department of Bioresources, University of Kashmir, India; c Molecular Biology Laboratory, Faculty of Veterinary Sciences and Animal Husbandary, SKAUST-K, Srinagar, India

**Keywords:** Adiponectin. Polycystic ovary syndrome, Gallic acid, CYP11a1, Cyp19a1

## Abstract

**Research question:**

Women are increasingly suffering from polycystic ovary syndrome (PCOS). Its pathophysiology is still unknown, though. The purpose of this study was to ascertain how gallic acid affected the pathophysiology of the ovary in an animal model of polycystic ovary syndrome. We also showed the potential mechanism of adiponectin involvement in endocrine metabolic changes in PCOS mice and the function of adiponectin, which appear to be frequent factors in PCOS.

**Design:**

Eighteen adult female Parkes strain mice (Age: 4–5 weeks) having body weight of 16–21 g were separated into three groups at random with 6 animals in each group as follows: Group I serving the control, received water and normal diet for 81 days; group II received oral gavage administration of letrozole (LETZ)(6 mg/kg b.w.daily), which was dissolved in 0.9 % NaCl solution for 21 days for the induction of PCOS and left untreated for 60 days; Group III received oral gavage administration of LETZ (6 mg/kg) for first 21 days followed by the administration of gallic acid (GA) (75 mg/kg b.w. orally daily) for 60 days.

**Results:**

We found LETZ-treated mice experienced PCOS-like symptoms, including increased Serum testosterone, LH/FSH ratio, body and ovarian weight, blood glucose, serum insulin levels and inflammatory Cytokines. We also found decreased serum estrogen, oxidant capacity and enzyme activity and altered ovarian cytoarchitecture, with multiple cysts apart from irregular estrous cycle. Furthermore, mRNA expression levels CYP11a, CYP19a1, Kitl, PTGS2 and Adipo R1 were decreased. Furthermore, LETZ-induced PCOS mice when treated with GA we observed decrease in testosterone, LH, LH/FSH ratio, blood glucose, serum insulin and inflammatory cytokines. GA treatment in PCOS mice also increased estrogen levels, and oxidant capacity as well as enzyme activity. Furthermore mRNA expression levels of CYP11a1, CYP19a1, KITL, PTGS2 and Adipo R1 were also increased in LETZ+GA treated mice. These changes were linked to lower levels of circulating adiponectin and were altered when the mice were administered with gallic acid.

**Conclusion:**

Gallic acid might be a potential therapy in treating PCOS by regulating endocrine and metabolic abnormalities that are brought on by a drop in adiponectin levels along with hyperandrogenism. Additionally, adiponectin seems to be a frequent factor in PCOS. In addition to reducing inflammation-related comorbidities linked to LETZ-induced PCOS, GA enhances mRNA expression levels CYP11a, CYP19a1, Kitl and PTGS2 and hence reduces endocrine and metabolic abnormalities.

## Highlights

• Adiponectin appears to be a common point between PCOS and its symptoms.
• Gallic acid increases mRNA expression of CYP11a1 and CYP19a1.
• Gallic acid increases ovarian anti-oxidant capacity and reduces inflammatory cytokines in letrozole-induced PCOS.
• Gallic acid increases ovarian proton pump ATPase enzymes activity in Letrozole induced PCOS.

## Graphical abstract

**Figure:**
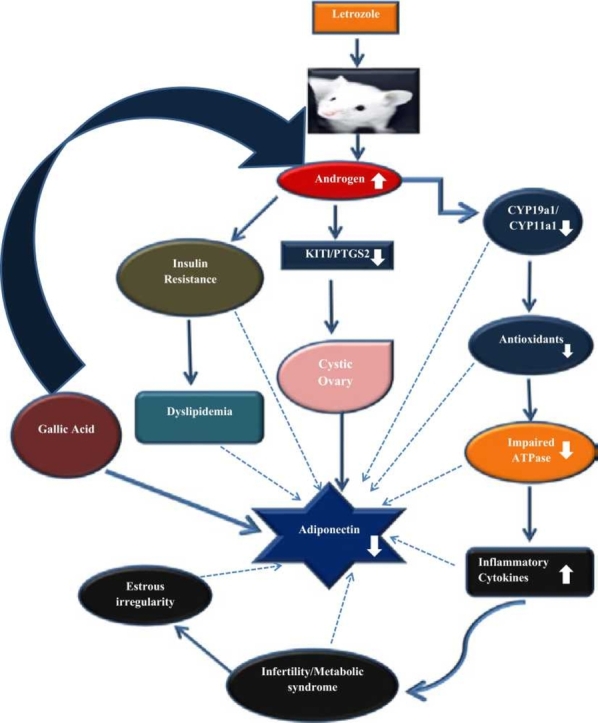

## 1 Introduction

A sexually active, non-contraceptive couple experiences infertility if they are unable to conceive naturally within a year of having unprotected sexual contact [1]. It has been estimated that between 20 % and 35 % of infertility cases are attributable to female factors, often resulting from ovulatory issues, which are typically exhibited by irregular or nonexistent menstrual cycles [3], [2]. High testosterone levels, menstrual irregularities and cystic ovaries are all symptoms of the complicated disease known as polycystic ovarian syndrome (PCOS). Polycystic ovaries are a physical manifestation of the condition, while hyperandrogenemia is primarily a biochemical one [4]. The chief manifestations of polycystic ovary syndrome caused by hyperandrogenism include decline in the ovarian reserve, poor quality of eggs, mitochondrial malfunction, anovulation and formation of cysts [4]. Microcysts in the ovaries, anovulation, and menstrual abnormalities are all symptoms of hyperandrogenism, a clinical feature of PCOS [5].

Obesity has been shown to be associated with between 44 % and 61 % of PCOS incidence, impacting infertility without PCOS, exacerbating the reproductive and metabolic symptoms of the condition [6], [7], [8]. Adiponectin is one of the proteins produced by an active endocrine, the adipose tissue [9], [10]. The crucial adiponectin not only reduces inflammation and prevents atherosclerosis but also regulates insulin, fat levels as well as blood sugar [11]. Due to its multidimer forms, adiponectin plays a variety of roles [12]. Nevertheless, its diminished secretion and the accompanying low levels of circulating adiponectin have been linked to metabolic issues including PCOS [13], [14]. Furthermore, it has been found that visceral adiposity and adipocyte mass, two significant risk factors for metabolic and related disorders, have inverse relationship with the circulating adiponectin [15], [9] , suggesting increasing visceral adiposity decreases its concentration. While some have suggested that adiponectin may be causal [16], and others identified adiponectin as a biomarker of insulin resistance (IR), despite years of study, specific function of adiponectin in metabolic diseases are still a mystery [17]. The adiponectin levels of blood have also been reported to be lower in PCOS patients, according to numerous researches. Adiponectin links metabolic disorders and insulin resistance [18], [19]. Recent studies [20], [21], [22] have raised a possibility that this adipocytokine may play a role in the to the metabolic anomalies connected to obesity and disorders associated with obesity, such polycystic ovarian syndrome. However, the exact nature of this adipocytokine function isstill unknown. In order to recognise and treat this complicated ovarian condition, therefore understanding how adiponctin contributes to the endocrine and metabolic problems associated with PCOS may be useful.

PCOS has been studied experimentally in rats using a variety of methods, including the injection of DHEA, excess prepubertal androgen, and estradiol valerate [13]. These models all produce PCOS, but none of them are completely convincing or precisely match the symptoms of real PCOS. The PCOS model caused by letrozole, a non-steroidal aromatase inhibitor, resembles human PCOS in many ways [23]. As was previously stated, hyperandrogenism is a major contributor to PCOS [24]. Aromatase (Cyp19a1) converts testosterone into estrogen in the granulosa cells of ovarian follicles. We gave letrozole to female mice in order to produce PCOS model that is comparable to PCOS of humans. Letrozole (LETZ) is a non-steroidal aromatase inhibitor that reduces production of estrogen in rats with symptoms similar to PCOS [25]. When the activity of the aromatase enzyme was inhibited, androgen accumulated, which caused an endocrine imbalance [25]. Hyperglycemia has been connected to letrozole induction, which has been connected to insulin resistance, hyperlipidemia, and metabolic syndrome [26], [27].

The use of human chorionic gonadotrophin (HCG) injections along with clomiphene citrate is one of the most well-liked treatments [28]. Patients with PCOS who use clomiphene report improved ovarian function, menstrual cycle, and glucose metabolism [29], [30]. Due to its molecular similarities to eestrogen compounds, clomiphene may have negative effects on endometrial thickness [31], [30]. Today doctors advise patients to adopt herbal medicines since they have fewer or no adverse effects because long-term use of chemical pharmaceuticals like metformin and other contraceptives can have a variety of bad effects [31], [29].

Gallic acid (GA), sometimes referred to as 3,4,5-trihydroxybenzoic acid, is one of the phenolic acids that is most commonly found in the plant kingdom. It is a crystalline substance that can be white or slightly yellow and finds extensive use in the food and pharmaceutical sectors. By using variety of chromatographic techniques, GA has been isolated from a variety of plant species, such as quercus and punica. However, from an industrial perspective, tannic acid is hydrolyzed to form GA using tannase, a glycoprotein esterase. [32]. Due to their ability to scavenge free radicals and act as antioxidants, GA and its derivatives, including, propyl gallate, tetradecyl gallate, hexadecyl gallate, lauryl gallate, and octyl gallate, can prevent the oxidation and rancidity of oils and fats. Consequently, they can be beneficial as food industry additives [33]. Numerous scientific studies have been done on these phytochemicals' biological as well as pharmacological activities, with a focus on their anti-inflammatory, anti-cancer, antioxidant, antimicrobial, cardioprotective, neuroprotective, and gastroprotective properties[33]. It has also been found that gallic acid restores hormonal imbalance in letrozole induced PCOS rats [34].

There are still very few studies on the impact of gallic acid on ovarian biology and polycystic ovarian syndrome. Therefore, the purpose of this study was to ascertain how gallic acid affected the pathophysiology of the ovary in an animal model of polycystic ovary syndrome. This study's main goal was to examine the effects of gallic acid on mice with letrozole-induced PCOS-related endocrine-metabolic disorders. The study also examined the impact of gallic acid on the circulating adiponectin and androgens in blood, as well as its function on PCOS-related endocrine and metabolic diseases.

## 2 Materials and methods

### 2.1 Animal treatments and tissue collection

18 adult parkes strain mice ( Age: av4–5 weeks) having body weight of 16–21 g were separated into three groups at random with 6 animals each as follows: Group I serving the control, received water and normal diet for 81 days; group II received oral gavage administration of letrozole (LETZ) (6 mg/kg bw) which was dissolved in availab0.9 % NaCl solution for 21 days for the induction of PCOS; GroupIII received oral gavage administration of LETZ (6 mg/kg) for 3 weeks followed by the administration of gallic acid (GA) (75 mg/kg bw orally daily) for 60 days. Samples of blood from all the mice were collected by puncturing retro-orbital venous sinus 24 h following 81 days of treatment. Serum was separated and used for biochemical and hormonal analysis. The mice were then cervically dislocated to death. For additional biochemical and gene expression research, the ovaries were removed from the body and fat tissue was cleaned. All experimental protocols were approved by Barkatullah University Bhopal's Institutional Ethics Committee (permission number 1885/GO/Re/S/16/CPCSEA/IAEC/BU/21).

### 2.2 Chemicals

Letrozole and gallic acid (GA) were purchased from Sun Pharma Company and Sigma eldrich respectively. Purchase order (492/biosc) from Clementia Biotech Delhi was used to purchase the Elisa kits (ELK Biotechnology Wuhan, China) for the hormonal analysis. Other substances employed in the investigation were analytical-grade substances.

### 2.3 Confirmation of PCOS induction

The estrous cycle was observed on daily basis, and vaginal smears were collected and spread out on a clean glass slide. These steps were carried out in compliance with Rotterdam standards (2003). After being fixed in methanol and given time to dry, cells were dyed using 0.76 g of giemsa [35]. In contrast to PCOS animals, which exhibited predominate leukocytes, which is the indication of diestrous phase (anovulation). The mice from group 1 serving as normal healthy control, displayed normal estrous cycle with the presence of proestrous, estrous, metestrous and diestrous phases. Additionally, histological analyses were performed 21 days after the oral gavage treatment letrozole, and they showed the presence of many cysts.

### 2.4 Biochemical analysis

#### 2.4.1 Analysis of hormones

Blood was collected by retro-orbital venous sinus puncture on 61st day of the experiment. Serum was then collected by centrifugation, stored as per manufacturer’s instructions until use. The competitive inhibition enzyme immunoassay approach was the foundation for the employed ELISA kits. The microtiter plate included in the kits has a particular protein pre-coated on it. Following the addition of standards or samples, a biotin-conjugated antibody for testosterone, LH, FSH, and estrogen was added to relevant microplate wells. Each microplate well was next added by an avidin conjugated to horseradish peroxidase (HRP), which was then incubated after the addition of TMB substrate solution. The kits' stop solution was used to halt the enzyme-substrate reaction, and an ELISA reader operating at a wavelength of 450 ± 10 nm was used to detect the colour change.

#### 2.4.2 Serum insulin and fasting blood glucose

Insulin levels in serum were tested using an ELISA kit acquired from ELK biotechnology, China. Fasting blood glucose was analysed by using commercially available kit.

#### 2.4.3 Serum Lipid profile

. Using readily accessible kits, total cholesterol (TC), triglycerides (TG), and high density lipoprotein (HDL) was colorimetrically assessed (Meril Diagnostics). While applying Friedewald's equation, low density lipoprotein and extremely low density lipoprotein were indirectly measured.

### 2.5 Antioxidant assay

#### 2.5.1 Lipid peroxidation assay

Malonaldehyde (MDA) content was used to gauge the extent of lipid peroxidation (LPO) in the ovary [36]. Utilizing a glass homogenizer, the ovaries were removed, and 10 % tissue homogenate was created in ice-cold normal saline. The sample was centrifuged for 10 min at 3000 rpm after the ovarian tissue had been homogenised. The supernatant was incubated for two hours in a millilitre at g g 37 °C. Following the correct mixing of each sample with 1 millilitre of 10 % tris hydrochloric acid (TCA), the samples were centrifuged at 2000 rpm for 0 minutesg g 5 min at 4 °C. Before being placed in boiling water bath for 10 min, 1 ml of supernatant was well mixed with an equivalent volume of 10%10 %0.67 % 2-thiobarbituric acid TBA. The samples were chilled and then diluted with 1 cc of distilled water (DW). A spectrophotometer was used to calculate the optical density (OD) at 535 nm. Data was calculated as nanomoles per gramme in moist tissue.

#### 2.5.2 Superoxide dismutase

Marklund & Marklund method, was used to assess the superoxide dismutase (SOD) activity [37]. Three minutes were utilised to test absorbance at 420 nm using 100 ml of pyrogallol and 2.9 ml of tissue homogenate supernatant (10 %). (0.2 mM). Activity of SOD was measured in units per gram of moist tissue.

#### 2.5.3 Determination of tissue catalase activity

Catalase activity was measured using the Sinha-described method [38]. The conversion of dichromate in acetic acid to chromic acetate under heat in the presence of H2O2 results in the formation of perchromic acid, an unstable intermediate, which serves as the basis for the process. The resulting chromic acetate is quantified by colorimetric measurement at 570–610 nm. Dichromate does not have an absorbance in this range, hence its inclusion in the assay mixture has no impact whatsoever on the colorimetric detection of chromic acetate. For varying lengths of time, H_2_O_2_ is divided by the catalase preparation. After heating the reaction mixture, termination of reaction was done at specific time reaction by adding dichromate/acetic acid mixture, the amount of water still present was evaluated by measuring chromic acetate by colorimetric analysis.

### 2.6 Cytokines

#### 2.6.1 Test procedure for plasma vascular endothelial growth factor (VEGF)

Sandwich-ELISA principle was employed in the ELISA kit. This kit's micro ELISA plate was pre-coated with a vascular endothelial growth factor-specific antibody (VEGF). The micro ELISA plate wells received standards or samples in addition to the particular antibody. After that, each microplate well received consecutive additions of an Avidin-Horseradish Peroxidase (HRP) combination and a biotinylated detection antibody specific for VEGF. Free parts were removed by washing. To each well, the substrate solution was added. The only wells that displayed blue coloration were those that also included VEGF, biotinylated detection antibody, and Avidin-HRP conjugate. By adding stop solution, the enzyme-substrate reaction was stopped, and the colour changed to yellow. At a wavelength of 450 nm, the optical density (OD) was measured spectrophotometrically. The relationship between the OD value and VEGF concentration is linear. It was feasible to determine the OD of the samples in relation to the standard curve how much VEGF was present in each sample.

#### 2.6.2 Test principle for tumor necrosis factor-alpha (TNF-ɑ)

Enzyme-Linked Immunosorbent Assay, also known as the ELISA kit, was used in the investigation. TNFɑ was injected into same wells that had previously undergone TNFɑ monoclonal antibody treatment. After incubation, an anti-mice TNF-biotin-conjugated antibody was added; this antibody binds to mice TNF-ɑ. Unbound biotin-conjugated anti-mice TNF-ɑantibody was eliminated during the washing phase that followed incubation. After that, streptavidin-HRP was used to bind to the biotin-conjugated anti-mice TNF-ɑ antibody. After incubation, washing was used to get rid of any unbound streptavidin-HRP. After that, a substrate solution was introduced, and as mice TNF-ɑ levels rise, the colour also does. The procedure was halted by adding an acidic stop solution, and absorbance was measured at 450 nm.

#### 2.6.3 Test principle tissue IL6

The ELISA kit used a technique known as the Sandwich-ELISA. An anti-IL6 antibody was used to pre-coat the micro ELISA plate in this assay. The micro ELISA plate wells included the particular antibody as well as standards or samples. Each microplate well was next treated with a biotinylated detection antibody specific for IL6, and then an Avidin-Horseradish Peroxidase (HRP) solution was added. Washing was used to take out the free pieces. Substratum solution was applied to each well. The only wells that displayed blue colour were those that also contained an Avidin-HRP conjugate, IL6, and a biotinylated detection antibody. By adding stop solution, which likewise caused the colour to change to yellow, the enzyme-substrate reaction was stopped. A wavelength of 450 nm was used to spectrophotometrically determine the optical density (OD). A linear association exists between the OD value and the VEGF concentration. It was feasible to determine the OD of the samples in relation to the standard curves how much IL6 was present in each sample.

### 2.7 Analysis of Ca2 + , Na+ /k + , & H ^+^ ATPase activity in the tissue of ovary

To a test tube, were added 0.5 ml of each of the compounds listed below: 0.35 M sodium chloride, 1.75 mM potassium chloride for the Na+ /K+ ATPase, and 0.5 ml of each of the following: 17.5 mM calcium chloride, 17.5 mM potassium chloride, 21.0 mM magnesium chloride, and 10 mM Tris HCl are required for the Ca2 + ATPase. It was given 0.2 ml of tissue homogenate and incubated for 60 min at 37 °C. By adding 0.8 ml of ice-cold, 10 % (w/v), trichloroacetic acid, the process was stopped (TCA). After that, it was centrifuged for 5 min at 4000 rpm at 4 °C for 20 min. Then, after 20 min at room temperature, 1 ml of twenty five percent ascorbic acid was added to 1 ml of supernatant. Then, a spectrophotometer was used to measure the absorbance at 725 nm in accordance with the procedures developed by Hjertén and Pan [39], [40] for the Na+ /K+ and Ca2 + ATPases, respectively. After that, we assessed enzymes by using Evans method [41]. In order to activate H+ ATPase, wait 10 min after adding 1 ml of 1.25 % Ammonium molybdate to 1 ml of the supernatant. The absorbance at 725 nm was measured using a spectrophotometer in accordance with [42], and the enzyme was similarly evaluated using Evans' after the addition of one ml of nine percent ascorbic acid [41]. Enzyme activity was calculated as 10^-3^ moles of pi per mg of protein per hour).

### 2.8 Reverse transcription and real-time PCR

As directed by the manufacturer, total RNA was extracted from the supernatants of ovary using TRI Reagent (Invitrogen), and cDNA was synthesised from 1 µg of total RNA using the cDNA Synthesis kit (Invitrogen). Real time PCR and SYBR green were used to accomplish RT-PCR. A standard curve was created for each gene with the associated expression levels in order to compute the mRNA values. The primers along with internal control actin used are listed in Table 1.

**Table 1:** List and sequence of primers used for the analysis of RT-PCR.

S.no	Gene	Forward primer (5ˈ –3ˈ)	Reverse primer (3ˈ –5ˈ)
1	**Kitl**	GGTAGCCAGGAGTTTGTTCT	TTGTGTGGCATAAGGGCT
2	**CYP11a1**	TCCTCAAAGCCAGCATCA	ATCTCGACCCATGGCAAA
3	**CYP19a1**	ATGTTCTTGGAAATGCTGAACCC	AGGACCTGGTATGAAGACGAG
4	**Ptgs2**	ACCTCTCTGAACTATGGTGT	TGCAGTCTGCTTTATGCG
5.	**AdipoR1**	CGC TTT CTG CGT ATC GTC TG	CCA ACC TGC ACA AGT TCC CTT
6.	**Actin**	TACGTCGCCCTGGATTTT	ATGAAAGAGGGCTGGAAGAG

### 2.9 Plasma adiponectin

Using elisa kits purchased from Elabscience Biotechnology Inc., the levels of adiponectin in the blood plasma were measured based on sandwich ELISA technique.

### 2.10 Ovarian histological evaluation

The ovaries were cut into 5-mm sections and overnight fixed in 10 % Bouin's fixative before being stained with hematoxylin and eosin (H& E).

### 2.11 Statistical analysis

ANOVA (one-way analysis of variance), followed by a post hoc analysis utilising Tukey's multiple comparison test, was used to determine the significance of the parameters. ANNOVA was performed by using Graph Pad prism. Data are presented as standard error of mean (±SEM). P values of 0.05, 0.01 and 0.001 were used to define statistically significant, more significant, and extremely significant, respectively.

## 3 Results

### 3.1 Effect of oral administration of Gallic acid (GA) on ovary and body weights in letrozole-Induced PCOS mice

**Fig. 1:**
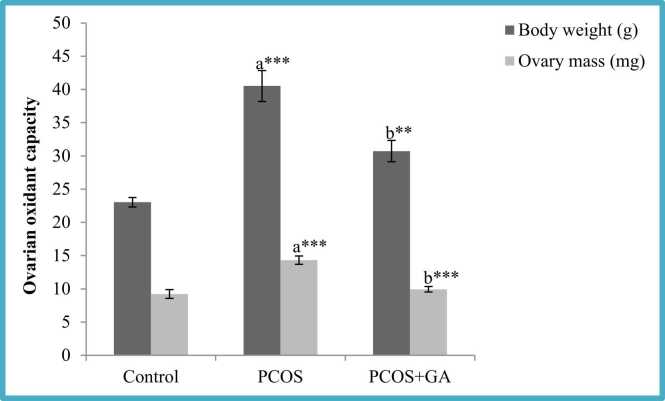
Effect of oral Gallic acid treatment on mass of body and ovary in mice with PCOS. All values displayed are expressed in mean±SEM with (n = 6 per group. Control: 0.9 % saline water: PCOS (LETZ 6 mg/kg); PCOS+ GA (Letz (6mgkg) and gallic acid (75 mg/kg); a= Control vs PCOS; b= PCOS vs PCOS+GA; ***p < 0.001, **P < 0.01.

The results revealed that letrozole administration produced a substantial rise (P < 0.001) in body weight as well as ovarian mass in comparison to control group, However, in the group administered with LETZ+GA we observed significant reduction (p < 0.001) in body weight and ovarian mass when compared to LETZ- induced PCOS group (see Fig. 1).

### 3.2 Effect of oral administration of Gallic acid (GA) on insulin and fasting blood glucose (FBG) in letrozole-Induced PCOS mice

**Fig. 2:**
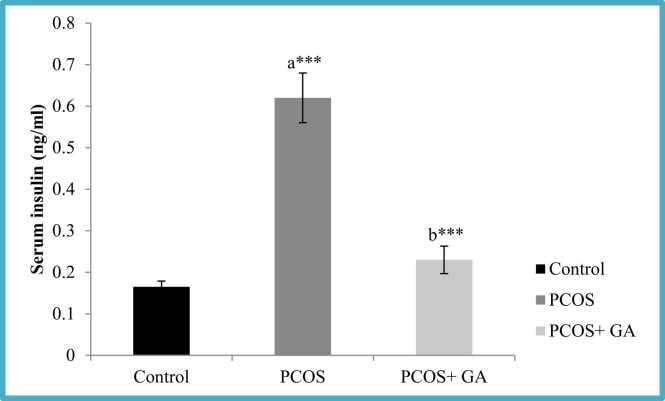
Effect of oral Gallic acid treatment on serum insulin levels in mice with PCOS. All values displayed are expressed in mean±SEM with n = 6 per group. Control: 0.9 % saline water: PCOS (LETZ 6 mg/kg); PCOS+ GA (LETZ: n6 mg/ kg and GA: 75 mg/kg); a= Control vs PCOS; b= PCOS vs PCOS+GA; * **p < 0.001.

The results confirmed that treatment of letrozole for 21 days resulted in significant rise (P < 0.001) in FBG as well as insulin levels as compared to the control group, while as, However, in the group administered with LETZ+GA, we observed significant reduction (P < 0.001) in the FBG and insulin levels as compared to the LETZ-induced PCOS group (Fig. 2; table2).

### 3.3 Effect of oral administration of Gallic acid (GA) on estrous cyclicity in letrozole-Induced PCOS mice

**Fig. 3:**
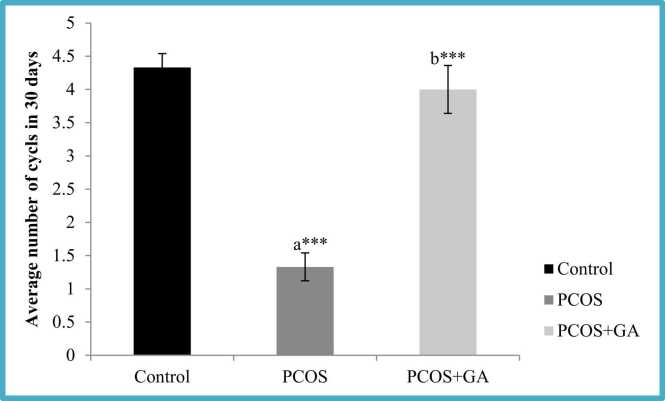
Effect of oral Gallic acid treatment on average number of cycles in mice with PCOS. All values displayed are expressed in mean±SEM with n = 6 per group. Control: Fig0.9 % saline water: PCOS (LETZ 6 mg/kg); PCOS+ GA (LETZ: 6 mg/kg and GA: ******75 mg/kg); a= Control vs PCOS; b= PCOS vs PCOS+GA; ***p < 0.001.

We observed LETZ-induced PCOS mice showed irregular estrous cyclicity wherein we observed a persistent diestrus condition which resulted in prolonged estrous cycles in comparison to the normal control mice. However, upon administration of GA, the estrous cycle was regularised resulting in the restoration of normal cycle length (Fig. 3).

### 3.4 Effect of oral administration of Gallic acid (GA) on serum testosterone, LH, FSH and estrogen levels in letrozole-Induced PCOS mice

**Fig. 4:**
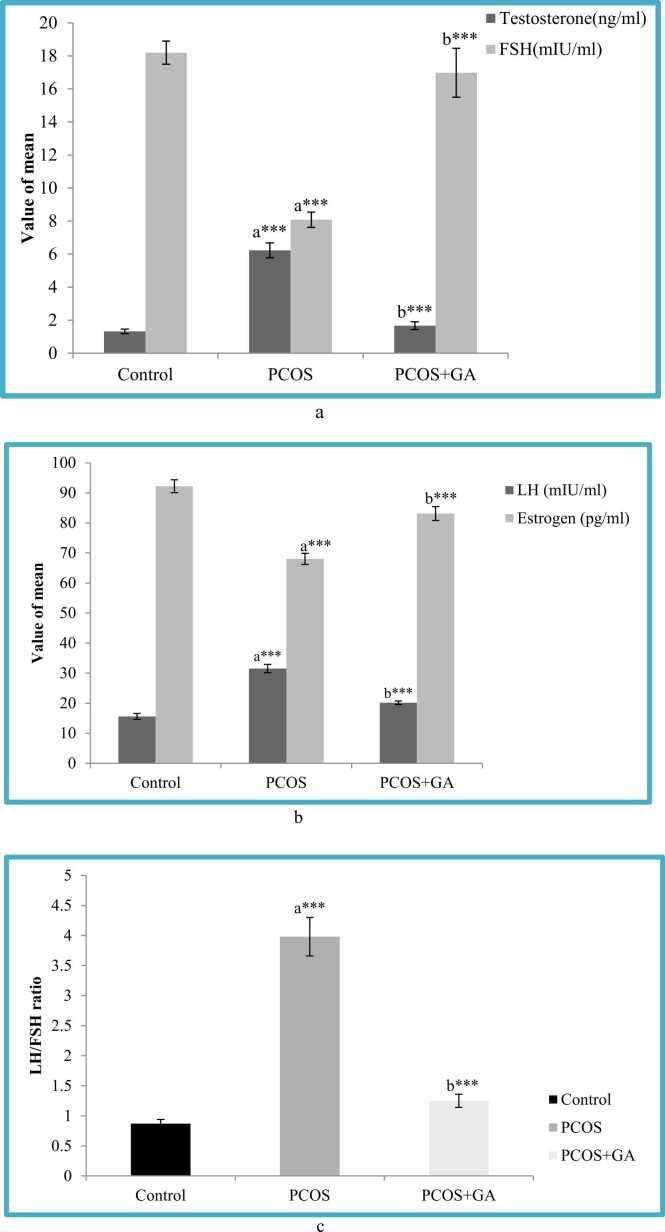
a. Effect of oral Gallic acid treatment on serum testosterone and FSH in mice with PCOS. All values displayed are expressed in mean±SEM with n = 6 per group. Control: 0.9 % saline water: PCOS (LETZ 6 mg/kg); PCOS+ GA (LETZ: 6 mg/kg and GA 75 mg/kg); a= Control vs PCOS; b= PCOS vs PCOS+GA; ***p < 0.001,. Fig. 4b. Effect of oral Gallic acid treatment on serum LH and esrtrogen in mice with PCOS. All values displayed are expressed in mean±SEM with n = 6 per group. Control: 0.9 % saline water: PCOS (LETZ 6 mg/kg); PCOS+ GA (LETZ: 6 mg/kg and GA: 75 mg/kg); a= Control vs PCOS; b= PCOS vs PCOS+GA; ***p < 0.001. Fig. 4c. Effect of oral Gallic acid treatment on serum LH/FSH in mice with PCOS. All values displayed are expressed in mean ± SEM with n = 6 per group. Control: 0.9 % saline water: PCOS (LETZ 6 mg/kg); PCOS+ GA (LETZ: 6 mg/kg and GA: 75 mg/kg); a= Control vs PCOS; b= PCOS vs PCOS+GA; ***p < 0.001,.

The results showed that LETZ- induced PCOS-mice showed significant increase m(p < 0.001) in serum testosterone, LH as well as LH/FSH levels, and substantial decrease m(p < 0.001) in estrogen and FSH as compared to the normal control. However, in the group administered with LETZ+GA, we observed significant reduction (P < 0.001) in serum testosterone, LH and LH/FSH levels as well as significant increase (p < 0.001) in estrogen and FSH levels as compared to the LETZ-induced PCOS group (Fig. 4a-c).

### 3.5 Effect of oral administration of Gallic acid (GA) on lipid profile in letrozole-Induced PCOS mice

**Fig. 5:**
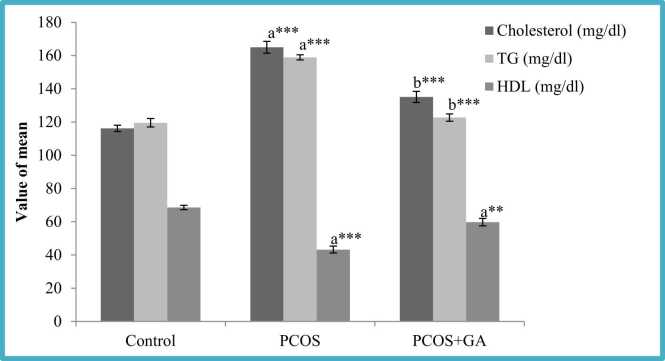
Effect of oral Gallic acid treatment on serum cholesterol, TG and HDL in mice with PCOS. All values displayed are expressed in mean±SEM with n = 6 per group. Control: 0.9 % saline water: PCOS (LETZ 6 mg/kg); PCOS+ GA (LETZ: 6 mg/kg and GA: 75 mg/kg); a= Control vs PCOS; b= PCOS vs PCOS+GA; (* **p < 0.001,.

Cholesterol, triglycerides (TG), LDL, and VLDL levels were found to be considerably higher (P < 0.001) in LETZ induced-PCOS mice compared to the control. We observed a substantial decrease (P < 0.001) in the LETZ+GA treatment group as compared to the LETZ-induced PCOS group. On the other hand, we discovered that HDL levels were significantly lower (P < 0.001) in LETZ-induced PCOS mice compared to the normal control, but after GA treatment, we noticed a considerable increase ((P < 0.001)) in these levels (Fig. 5; Table 2).

**Table 2:** Effect of oral Gallic acid treatment on Fasting blood glucose, LDL, VLDL and estrous cycle in mice with PCOS.

Group	Fasting blood glucose (mg/dl)	Low density lipo-protein (LDL) (mg/dl)	Very low density lipo-protein (VLDL) (mg/dl)
Control	107.46 ± 1.51	23.68 ± 2.96	23.91 ± 0.26
PCOS	157.52 ± 1.99^a***^	64.60 ± 2.53^a***^	31.78 ± 0.41^a***^
PCOS+GA	122.36 ± 3.31^b***^	50.74 ± 2.71^b***^	24.54 ± 0.45^b*^

All values displayed are expressed in mean±SEM with n = 6 per group. Control: 0.9 % saline water: PCOS (LETZ 6 mg/kg); PCOS+ GA (LETZ: (6mgkg) and GA: 75 mg/kg); a= Control vs PCOS; b= PCOS vs PCOS+GA; 7*p < 0.05, ***p < 0.001,.

### 3.6 Effects of Gallic acid (GA) on ovarian oxidant capacity in letrozole -Induced PCOS mice

**Fig. 6:**
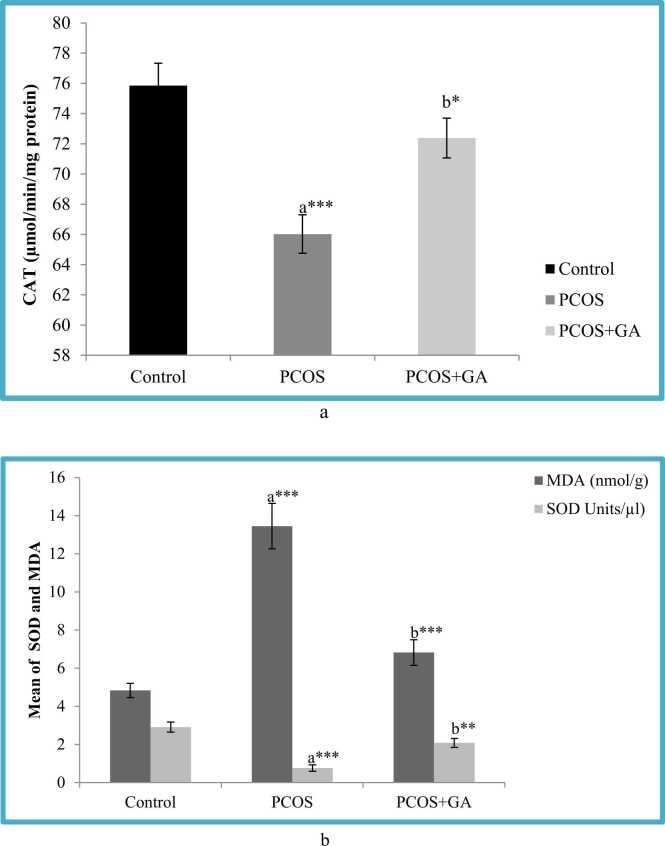
a Effect of oral Gallic acid treatment on CAT activity in ovary in mice with PCOS. All values displayed are expressed in mean±SEM with n = 6 per group. Control: 0.9 % saline water: PCOS (LETZ 6 mg/kg); PCOS+ GA (LETZ: 6mgkg and GA: 75 mg/kg); a= Control vs PCOS; b= PCOS vs PCOS+GA; * **p < 0.001, *p < 0.05. Fig. 6b. Effect of oral Gallic acid treatment on MDA and SOD activity in ovary in mice with PCOS. All values displayed are expressed in mean±SEM with n = 6 per group. Control: 0.9 % saline water: PCOS (LETZ 6 mg/kg); PCOS+ GA (LETZ: 6 mg/kg and GA: 75 mg/kg); a= Control vs PCOS; b= PCOS vs PCOS+GA; ***p < 0.001, **p < 0.01.

According to our findings, LETZ- induced PCOS-mice showed significantly reduced (p < 0.001) levels of SOD and CAT in the ovary when compared to control mice, However, in the group administered with LETZ+GA,we observed significant increase(P < 0.001) in the SOD and CAT levels as compared to the LETZ-induced PCOS untreated group. Moreover MDA being the end product of the lipid peroxidation reaction which was found significantly increased (P < 0.001) in the LETZ-induced PCOS mice, however in the group which was treated with LETZ+GA we observed significant drop (p < 0.001) in the MDA levels as compared to the LETZ-induced PCOS group (Fig. 6a-b).

### 3.7 Effects of oral administration of GA on ovarian proton pump ATPase enzymes activity (Na+/ K+, Ca_2_ +and H+ ATPase) activity in letrozole -Induced PCOS mice

Letrozole (LTZ) administration resulted in significant decrease (p < 0.001) in the activity of the proton pump enzyme as compared to the control group. However, we observed that the group administered with LETZ+GA the activity of the proton pump enzymes were significantly increased (p < 0.01) (Table 3).

**Table 3:** Effect of oral Gallic acid treatment on activity of ATPase enzymes (Piµmol/mgprotein/hr/^10–3^) in mice with PCOS.

Group	Na^+^/K^+^ ATPase	Ca_2_^+^ ATPase	H^+^ ATPase
Control	0.23 ± 0.02	3.27 ± 0.09	0.19 ± 0.01
PCOS	0.13 ± 0.01^a***^	2.08 ± 0.07^a***^	0.11 ± 0.008^a***^
PCOS+GA	0.19 ± 0.004^b*^	3.24 ± 0.11^b***^	0.18 ± 0.004^b**^

All values displayed are expressed in mean±SEM with n = 6 per group. Control: 0.9 % saline water: PCOS (LETZ 6 mg/kg); PCOS+ GA (LETZ: 6 mg/kg and gallic acid: (75 mg/kg); a= Control vs PCOS; b= PCOS vs PCOS+GA; *p < 0.05, **p < 0.01 and ***p < 0.001.

### 3.8 Effects of oral administration of GA on follicular phase transcriptional markers in the ovary of letrozole -induced PCOS mice

**Fig. 7:**
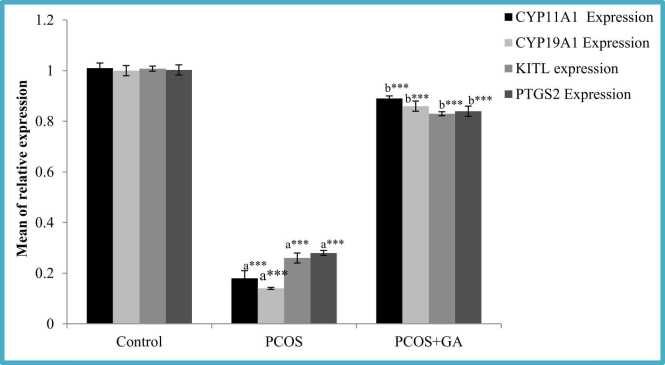
Effect of GA on CYP11a1, CYP19a1, PTGS2 and KITL, expression in mice with PCOS. All values displayed are expressed in mean±SEM with n = 6 per group. Control: 0.9 % saline water: PCOS (LETZ 6 mg/kg); PCOS+ GA (LETZ: 6 mg/kg and GA: 75 mg/kg); a= Control vs PCOS; b= PCOS vs PCOS+GA; * **p < 0.001.

We found LETZ- induced PCOS mice had decreased (p < 0.001) Kitl and PTGS2 mRNA expressions when compared to normal control; however, in the group treated with LETZ+GA these were significantly increased (p < 0.001) when compared with PCOS group without therapy (Fig. 7).

### 3.9 Effect of oral administration of GA on steroid synthesis-related genes in letrozole- induced PCOS mice

Two enzymes involved in the steroid biosynthesis pathway, were evaluated. In this study, we discovered that CYP11a1 and CYP19a1 mRNA expression levels were less (p < 0.001) in letrozole treated in comparison to normal control. However, in the group treated with LETZ+ GA we observed significant increase ((p < 0.001) in the levels of CYP11a1 and CYP19a1 expression in comparison to LETZ-induced PCOS group (Fig. 7).

### 3.10 Effects of oral administration of GA on plasma inflammatory cytokines (Tumor necrosis Factor- alpha and Vascular Endothelial Growth Factor and IL6) concentrations in Letrozole-Induced PCOS mice

**Fig. 8:**
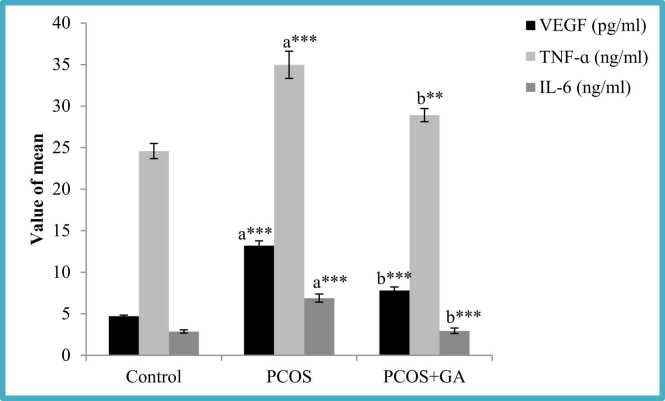
Effect GA on plasma TNFα, VEGF and IL-6 mice with PCOS. All values displayed are expressed in mean±SEM with n = 6 per group. Control: 0.9 % saline water: PCOS (LETZ 6 mg/kg); PCOS+ GA (LETZ: 6 mg/kg and GA: 75 mg/kg); a= Control vs PCOS; b= PCOS vs PCOS+GA; * **p < 0.001.

The result of this study confirmed letrozole administration resulted in the significant rise (p < 0.001) in TNF-ɑ, VEGF and IL-6 levels as compared to normal mice. However, the group which was administered with LETZ+GA, observed a significant decrease in their levels in comparison to LETZ-induced PCOS group (Fig. 8).

### 3.11 Effects of GA on plasma adiponectin and Adipo R1 expression in the ovary of letrozole-Induced PCOS mice

**Fig. 9:**
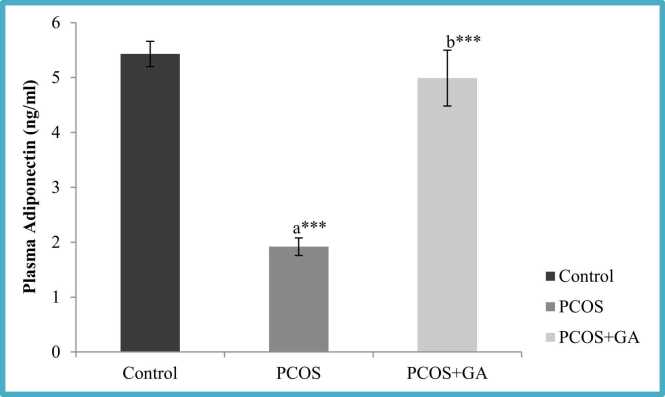
Effect GA on plasma adiponectin mice with PCOS. All values displayed are expressed in mean±SEM with n = 6 per group. Control: 0.9 % saline water: PCOS (LETZ (p&6 mg/kg); PCOS+ GA (LETZ: 6 mg/kg and GA: 75 mg/kg); a= Control vs PCOS; b= PCOS vs PCOS+GA; * **p < 0.001.

**Fig. 10:**
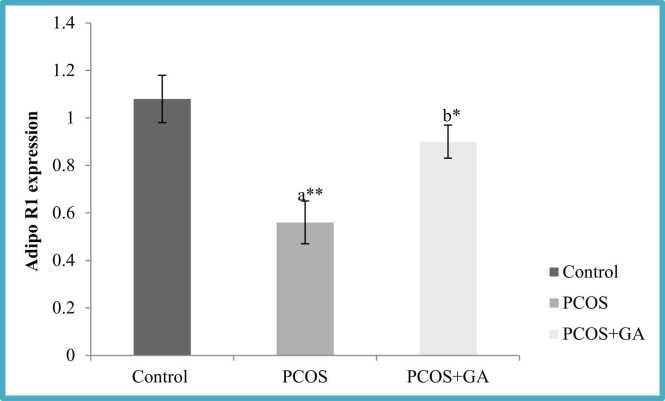
Effect GA on plasma ADIPOR1 expression of mice with PCOS. All values displayed are expressed in mean±SEM with n = 6 per group. Control: 0.9 % saline water: PCOS (LETZ 6 mg/kg); PCOS+ GA (LETZ: 6 mg/kg and GA: 75 mg/kg); a= Control vs PCOS; b= PCOS vs PCOS+GA; * *p < 0.01, *p < 0.05.

We observed letrozole administration resulted in the significant decrease (p < 0.001) in the adiponectin levels in comparison to the normal control. However, after administration of GA, we found there was significant decrease (p < 0.001) in the levels of adiponectin in comparison to the PCOS untreated group. Similarly AdipoR1 expression was significantly reduced (p < 0.01) in PCOS mice which was increased significantly when PCOS mice were given GA (Fig. 9, Fig. 10).

### 3.12 Effect of oral administration of GA on ovarian histology in letrozole-induced PCOS mice

We observed that letrozole administration resulted in the degeneration of corpus luteum, with increased number of cystic follicles when compared with the normal control. However, following GA delivery, ovarian tissue was preserved and exhibited healthy follicles with decline in cystic follicles (Fig. 12).

**Fig. 11:**
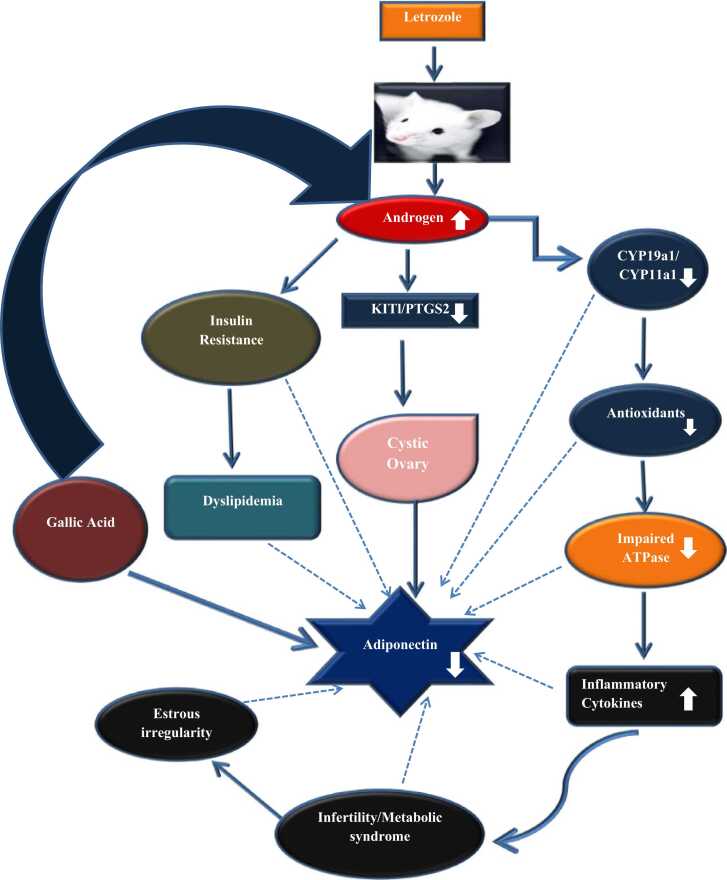
Figure shows the effect of Gallic acid on endocrine and metabolic abnormalities in PCOS and possible involvement of Adiponectin.

**Fig. 12:**
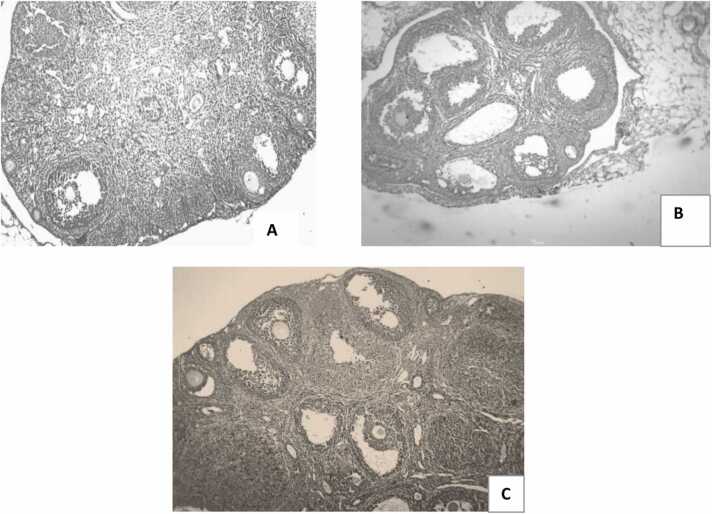
Ovarian photomicrographs from each experimental group (H&E, magnificent x10): (A) control ((B) PCOS, C) PCOS + GA.

## 4 Discussion

A complicated and polygenic condition, polycystic ovarian syndrome places a heavy financial burden on both individuals and society [43]. In addition to other difficulties like cardiovascular issues, insulin resistance apart from infertility clinical symptoms of PCOS also include acne, hirsutism, and abdominal obesity which are associated with elevated androgens leading women’s to depression as well as anxiety. Attention should be paid to novel pharmaceutical techniques with fewer side effects.

We found that gallic acid (GA) reverses the metabolic as well as endocrine abnormalities which are linked to LETZ-induced PCOS by regulation of androgen and adiponectin circulation. Using experimental female mice of parkes strain, we were able to confirm that letrozole induces PCOS, characterised by obesity and ovarian cysts. In addition to these elevated metabolic and circulatory markers, like dyslipidemia and elevated insulin levels. LETZ-induced PCOS mice are linked to elevated lipid peroxidation (MDA), impaired cellular antioxidant capacity in the ovary, greater levels of inflammatory cytokines, and decreased levels of adiponectin. These alterations were attenuated following GA treatment. (Fig. 11).

Through vaginal smears [35], which showed predominate leukocytes in letrozole groups indicating continuous diestrous phase, PCOS induction was confirmed in the current experiment. Notably, letrozole prevents androgens from being converted to estrogens, which causes hyperandrogenism [44]. Higher levels of androgen result in the estrous cycle dysregulation, increased reproductive organ and body weight. [5]. In line with earlier research, we found LETZ-treated mice had higher body and ovarian mass as well as anomalies in the estrous cycle. Following GA administration, there was a normal estrous cycle and a reduction in body weight as well as in the weight of ovary.

Women with polycystic ovarian syndrome, experience metabolic and associated problems which include IR, poor GT (glucose tolerance), and obesity apart from type 2 diabetes have been documented earlier [6], [45]. Results from this corroborated with previous findings in that LETZ-induced PCOS in mice resulted in higher insulin and fasting blood glucose levels, demonstrating insulin resistance and hyperglycemia, two key characteristics of metabolic diseases (MD’S) [46], [45]. In addition, hyperinsulinemia as well as hyperglycemia are crucial metabolic incidents that set off an inflammatory cascade and oxidative stress, particularly in metabolic syndrome, can contribute to target organ dysfunction, including ovarian dysfunction, both structurally and functionally. Similar to this, decreased insulin levels as observed animals with LETZ-induced PCOS may also have an impact on the activities of metabolic tissues mediated by insulin which include hepatic, skeletal muscle, adipose tissues leading to metabolic anomalies in tissue of ovary resulting in disrupted glucose and lipids. Additionally,past research have shown that insulin resistance causes an increase in adiposity, a significant contributing factor to obesity in metabolic and related illnesses [47], [48], [49].

There is evidence that ovarian tissues are stimulated to create androgen through inhibition of aromatase action by free insulin growth factors, insulin resistance, and the resulting hyperinsulinemia [50], [9]. We found that letrozole-treated mice had raised blood levels of testosterone and are consistent to earlier research [51], [50]. In the present study, however, testosterone levels were lowered by GA treatment. Increased levels of testosterone in the blood may partially be responsible for decreased IS(insulin sensitivity)which results in decreased peripheral glucose clearance, which further results in elevated glucose levels in animals with PCOS caused by LETZ. The current study also showed a significant rise in the LH/FSH ratio, which suggests a coping mechanism against the inhibition of estrogen synthesis and estrogen circulation which in turn has an impact on metabolic metrics, particularly IR [52]. Findings of this study therefore confirm as well as suggest that endocrine and metabolic disorders both contribute to the pathogenesis of PCOS. Additionally, they show that endocrine and metabolic variables can interact in a vicious cycle, which provides the physiological underpinnings for complex nature of PCOS and its clinical manifestation.

Hyperandrogenism as well as hyperinsulinemia in PCOS patients cause adipocytes to elevate catecholamine-induced lipolysis, which leads to elevated serum free fatty acid levels and, consequently, dyslipidemia [53]. As a result, the liver produces more free fatty acids, which raises blood levels of VLDL and TG [54]. In this study we found that, mice given letrozole had elevated TC, TG, LDL, VLDL levels and lower levels of HDL compared to the control. However, GA administration showed a substantial decline in these levels, furthermore levels of LDL levels were increased. Gallic acid was found to have a similar impact in previous study [55].

Additionally, past research has shown a connection between oxidative stress and dyslipidemia [57], [56]. In the current study we observed increase in the levels of MDA which in turn resulted in decreased CAT and superoxide mutase (SOD) in LETZ- induced PCOS, resulting in oxidative stress (OS). In this investigation, we found that the oral gavage treatment of gallic acid returned MDA, SOD, and CAT levels to normal, which are consistent with earlier studies the benefits of gallic acid in preventing oxidative stress from doing harm [58]. Additionally, the present study's findings showed that LETZ-induced PCOS animals had elevated levels of inflammatory cytokines, such as IL-6 and TNF-α in comparison to the control group. According to [54], the angiogenic factor VEGF is essential for pathological, physiological, and developmental angiogenesis. Oxidative stress sets up a pro-inflammatory condition that, in a feedback loop, causes insulin resistance as well as hyperandrogenism [43]. As a result, it is reported that PCOS women secrete more VEGF [59]. The mechanism is explained by the presence of androgen receptor (AR) binding sites in the VEGF promoter region. When androgens attach to these places, the VEGF gene is put into action [60]. In addition, soluble VEGF receptors decrease in PCOS women's blood, increasing the bioavailability of VEGF, as demonstrated by [59]. These results are consistent with the letrozole group's elevated VEGF levels that were noticed. In addition, when letrozole and GA were delivered together, the levels of TNF-, IL-6, and VEGF were lower than they were in the letrozole-only group as shown in Fig. 7.

By activating protooncogene and silencing antitumor genes, OS plays important role in earlier stages of tumour conversion and tumour aetiology [61]. Because of this, increased OS in polycystic ovarian syndrome may increase the risk of cancer and may lead to genetic instability as well. Inflammation, obesity, and hyperandrogenism—which are the prevalent symptoms and putative causes of ovarian dysfunction in PCOS patients—have been shown to be substantially correlated with oxidative stress. Reduced proton pump (ATPase) activity has been linked by various studies to problems with female reproduction. Impaired ATPase activity has been linked to oxidative stress, according to some research [62], [63]. Membrane lipid peroxidation, and inflammation as well as free radicals have all been found to greatly increase the susceptibility of ATPase to these conditions. Lipid peroxidation (MDA) was found to selectively change the activity of the, calcium ATPase, Mg^+^ ATPase as well as Na+ /K+ -ATPase [64]. The fact that Ca_2_ + , H+ - and Na^+^/K^+^ ATPase activity is impaired may also be explained by the fact that reactive oxygen species may considerably raise levels of beneficial antioxidants [65], [66] The group administered with GA and letrozole showed a significant increase H+ ATPase, Na+ /K+ as well as Ca2 + ATPase in comparison to letrozole group. Metabolic substrates like glucose and amino acids can be transported in the second step to proton pumps, which maintain constant trans-membrane ion gradients. Movement of the Na^+^, Ca2 + , K+ , and Mg2 + ions across the membrane is made possible by the ATPases that preserve the ionic gradient across the cell membrane, membrane potential, and osmotic equilibrium.

Kitl and Ptgs2, two genes associated with foliculogenesis, contribute to the development of oocytes through the Ptgs2 and KIT/KITL pathways [67]. In letrozole-induced PCOS mice and letrozole-coadministered GA animals, transcription of both genes was decreased and elevated, respectively. Furthermore, letrozole-induced rat ovaries had a thin follicular layer and more cysts than control rat ovaries in the ovarian tissue micrograph. On the other hand, follicular cysts and some corpus luteum were reduced in mouse ovaries that had received GA treatment. Both findings suggest that GA normalises follicular development and ovarian cysts. Furthermore, prior studies showing lower expression of aromatase enzyme and estrogen release in granulosa cells of DHT -treated rats were in agreement with the decrease of CYP19A1 in ovaries of LETZ treated mice [68]. In addition, mice given GA coupled with letrozole had higher levels of steroid synthesis-related gene mRNA expression, including Cyp19a1, required in converting androstenedione into estrogen. Under a microscope, the ovary of the letrozole-exposed mice can be observed to have greater cystic follicles, and no corpus luteum. As GA decreased levels of MDA increased cellular antioxidant capacity, tumor necrosis factor and IL6, ovarian tissue with healthy follicles and no cystic follicles was retained.

The considerable drop in adiponectin levels as well as Adipo R1 expression in LETZ-induced PCOS is especially noteworthy. mice compared to the control group because this data partially supports earlier research that adiponectin is a marker of IR in patients experiencing PCOS [9], [17]. Current investigation also found significantly decreased adiponectin levels in LETZ-induced PCOSmouse, along with changes in endocrine and metabolic factors like circulating testosterone, the LH/FSH ratio, blood sugar, insulin sensitivity, lipid profile, oxidative stress, pro-inflammatory biomarkers and ovarian tissue histology. According to various studies MD’s and related syndromes have reduced adiponectin concentrations [69], [11]. It was demonstrated that adiponectin, is a metabolic agent influencing steroidogenesis as well as gametogenesis and may also have an impact on synthesis of GnRH along with gonadal function besides its metabolic effects in numerous in vitro studies [70], [71]. Adiponectin has been connected to follicular dominance and oocyte competency in rats because there is a link between the ovarian cells of the dominant follicle's adiponectin transcript and the amount of estradiol in the follicular fluid [72].

Recombinant adiponectin has also been observed to boost the release of hormones when used at physiological dosages (5–10 g/ml), particularly estrogen, in IGF1-stimulated cells in rodents and women [72]. The results of our study suggest that adiponectin share certain characteristics with endocrine as well as metabolic variables in PCOS. Drop in its concentration may be a contributing cause to infertility in metabolic diseases like polycystic ovarian syndrome. Therefore, increase in its levels in PCOS patients may be a therapeutic approach for treating metabolic diseases and perhaps even partially endocrine issues.

## 5 Conclusion

A mice model of PCOS using an oral gavage treatment of letrozole was created to create a model with symptoms resembling those of women with the condition. Aditionally we observed amleorative role of gallic acid on PCOS using an in vivo PCOS model. According to the findings of the current investigation, GA might be a promising therapy in treating PCOS as it showed beneficial effect on hormonal imbalance, estrous irregularities part from other reproductive abnormalities that are brought on by a drop in adiponectin levels along with hyperandrogenism. Additionally, adiponectin seems to be a frequent factor in PCOS. In addition to reducing inflammation-related comorbidities linked to LETZ-induced PCOS, GA enhances mRNA expression levels CYP11a, CYP19a1, Kitl and PTGS2 and hence reduces endocrine and metabolic abnormalities.

## CRediT authorship contribution statement

**Mohd Zahoor ul haq Shah:** Conceptualization, Writing – original draft, Formal analysis, Resources, Investigation. **Vinoy kumar Shrivastava:** Supervision, Writing – review & editing. **Meenakshi Soni:** Writing - review & editing. **Ahmad Mir:** Writing – review & editing. **Showkeen Muzamil:** Writing – review & editing..

## Declaration of Competing Interest

The authors declare that they have no known competing financial interests or personal relationships that could have appeared to influence the work reported in this paper.

## Data Availability

Data will be made available on request.
